# Effects of Golden Plaster on Knee Osteoarthritis: A Multicenter Randomized, Double-Blind, Placebo-Controlled Trial

**DOI:** 10.1155/2022/4205648

**Published:** 2022-02-02

**Authors:** Yan Chen, Jintao Liu, Xiaofeng Li, Dezhi Tang, Xiaoqin Jin, Zhigang Zhang, Wanbo Ji, Shuai Tao, Hong Jiang

**Affiliations:** ^1^School of Rehabilitation Medicine, Henan University of Chinese Medicine, Zhengzhou 450046, China; ^2^Suzhou Hospital of Traditional Chinese Medicine, 889 Wuzhongxi Road, Suzhou 215009, China; ^3^Longhua Hospital affiliated to Shanghai University of Traditional Chinese Medicine, 725 Wan-Ping South Road, Shanghai 200032, China; ^4^Zhangjiagang Hospital of Traditional Chinese Medicine, Zhangjiagang 215600, China; ^5^Taizhou Hospital of Traditional Chinese Medicine, Taizhou 225314, China

## Abstract

**Background:**

Golden plaster is the preferred and most commonly used in China for pain reduction in patients with knee osteoarthritis (OA). However, there was no evidence-based medical evidence about its effect in relieving pain of knee OA patients. Here, a multicenter randomized, double-blind, placebo-controlled trial was performed to evaluate the efficacy and safety of golden plaster for the improvement of pain relief and function's obstacle in patients with knee OA.

**Methods:**

320 patients with knee OA were enrolled at four hospitals and randomly divided into the treatment group and the control group with 160 subjects in each group. Patients in treatment group were treated with golden plaster, and those in control group with placebo plaster. The study cycle in both groups was 21 days. Patient visits were documented before treatment and 7-, 14-, and 21-day follow-ups after treatment. The outcomes included VAS score, WOMAC score, and adverse events.

**Results:**

Compared to the control group, the VAS score in the treatment group was significantly decreased after treatment with golden plaster for 7 days, 14 days, and 21 days. Compared to the control group, the WOMAC score in the treatment group was significantly decreased 14 days and 21 days. The incidence rate of adverse events had no statistical difference between both the groups.

**Conclusions:**

In conclusion, our study, for the first time by carrying on the double-blind and placebo-controlled randomized trial, showed that golden plaster can effectively alleviate the pain of knee and improve the physical function in the patients with knee OA. This trial is registered with ChiCTR-TRC-13003418.

## 1. Introduction

Osteoarthritis (OA), a degenerative joint disease, is a common health problem in an aging society [1, 2]. The prevalence of OA in people over 45 years of age is 28%, and the symptoms worsen with age [3]. Knee OA is common in the elderly frequently occurring disease, seriously affecting people's health and normal life. The purpose of OA therapy is to reduce pain, improve function, and improve the quality of life of the patients. The nonsurgical treatment of OA includes drug and physical therapy [4–7]. Drugs used to treat knee osteoarthritis include oral analgesics, acetaminophen, ibuprofen, and traditional Chinese medicines (TCM) [8, 9]. In addition, proper exercise can maintain the range of motion of the joint [10–13]. Acupuncture and massage therapy are also used in the treatment of OA [14, 15]. However, the long-term analgesic effects of these therapies have not been fully demonstrated.

Complementary and alternative medicine therapies have been used for many years to relieve pain in patients with knee OA [16–19]. Plaster is a very important Chinese medicine therapy; it has the function of activating blood circulation and removing stasis, reducing swelling, and relieving pain [20, 21]. Among Chinese medicine therapy, gold plasters are effective in relieving pain in patients with knee osteoarthritis with few side effects.

At present, golden cream is mainly used in clinical treatment of bone and joint diseases, such as fracture, osteoarthritis, and rheumatoid arthritis. Modern research shows that golden cream has the function of promoting blood circulation, removing blood stasis, reducing swelling, and relieving pain and can accelerate the repair of damaged tissues [18, 19]. Since the placebo-control group was not used, the previous studies were lacking high quality of evidence-based medical evidence. Therefore, in this study, we performed a randomized, double-blind, placebo-controlled trial for the first time to evaluate the clinical efficacy and safety of golden plaster for therapy in patients with knee OA.

## 2. Methods

### 2.1. Study Design

The study was a multicenter, randomized, double-blind, placebo-controlled trial. The protocol was registered on the official website of China clinical trial center http://www.clinicaltrials.org. The subjects of this clinical trial were from Suzhou Hospital of TCM, Longhua Hospital affiliated to Shanghai University of TCM, Zhangjiagang Hospital of TCM, and Taizhou Hospital of TCM. Ethics committees at the four hospitals approved the study. All subjects signed written consent forms before the study began. The approval number of ethic approval was 2013LCSY0618.

### 2.2. Inclusion Criteria

The following were the inclusion criteria [22]: between 45 and 79 years old; pain in patients with knee osteoarthritis was at least 20 millimeters on a 100-millimeter Visual Analog Pain Scale (VAS); rheumatologists assessed symptoms in patients with knee OA based on the American College of Rheumatology criteria [23]; ability to read and speak; ability to understand learning requirements; and willing to cooperate with learning guidance.

### 2.3. Exclusion Criteria

The following were the exclusion criteria [22]: psoriatic arthritis, cancer, severe heart or kidney disease, severe trauma to knee joint, including severe ligament or meniscus injury within one year before the study, and allergic to plasters.

### 2.4. Recruitment

Participants were recruited by recruitment information posted in newspapers on hospital websites and bulletin board. All participants were evaluated with baseline data prior to randomization. When participants met inclusion criteria and signed informed consent, they were randomized.

### 2.5. Randomization

Participants' data were registered into a database for random grouping by the teletherapist. When the participants were ready to be randomized, the teletherapist typed “yes,” the randomization program automatically displayed the group and number of participants (treatment group and control group). The random lists were generated by a computer and hidden by a senior data manager who was not involved in the study. According to the CONSORT guidelines, this information was confidential and was not shared with the research center. The study was a randomized, double-blind trial, and all results were assessed by a research assistant who was not aware of the grouping.

### 2.6. Intervention

The study was consistent with the Helsinki Declaration and approved by the appropriate institutional review committee. All drugs were administered externally for 3 weeks. Our study nurses, who had been trained before the study began, instructed participants in the proper use of golden plaster. Double-blind design was adopted for this study: the therapists, subjects, investigators, and statisticians were not aware of treatment assignments. The randomization of participants was carried out by random number generator. Participants were visited at baseline, 7, 14, and 21 days after treatment. Participants were assessed on all four visits.

Patients in the treatment group were treated with golden plaster. Herbs in the golden plaster (Table 1) were uniformly provided by Shanghai Huayu Chinese Herbs Co., Ltd., China, and were accredited by a pharmacognosist according to standard protocols, which were provided by Suzhou Hospital of Traditional Chinese Medicine. The accessories used to make golden plaster were sesame oil, beeswax, and glycerinum. Golden plaster was prepared according to standard methods of Chinese Pharmacopoeia (China Pharmacopoeia and Committee, 2000). The herbs of golden plaster were dried, crushed into fine powers, and filtered through an 80-mesh sieve. Sesame oils after heat refining were mixed with the filtered beeswax and then added into glycerin. Herbs powers were slowly sprinkled into the ointment matrixes, stirred until condensation, kept in bags, and divided on 10 × 20 cm tissue papers. Placebo pastes were identical to golden pastes in terms of texture, size, color, and odor. While treating, the paste was directly contacted with the skin around knee joint, dressed with a layer of cotton, and fixed with the bandage externally. The dressing changed every 7 days, and one period of treatment was 21 days. The control plaster is made by the same drug of golden plaster with the dosage of 10%. The control plaster is added the dark yellow pigment properly to make it look the same as golden plaster, and the control plaster was made as the same characters, pack, specification, size, color, and odor as golden plaster. In addition, the usage of the control plaster is also the same as golden plaster.

The products used were packaged and labeled by the Suzhou Hospital of TCM to ensure that researchers and participants were not aware of the treatment assignments according to the current regulatory requirements. Patients were not allowed to take other drugs during the study.

### 2.7. Outcome Measures

#### 2.7.1. Primary Outcome Measure

The VAS was adopted to evaluate the efficacy, which was a pain scale from 0 mm (no pain) to 100 mm (most painful) [24]. Operationally, the VAS score was a horizontal line of 100 mm in length. The patient was marked on this line segment according to the degree of pain. The VAS score was determined by measuring the length from the left end of the line segment to the patient marker. The VAS scores were assessed during all visits to the patients (baseline and 7, 14, and 21 days after treatment).

#### 2.7.2. Secondary Outcome Measure

Western Ontario and McMaster University Osteoarthritis Index (WOMAC) score was also measured in this study. WOMAC score is a recognized self-rating scale about knee osteoarthritis. The WOMAC score is made up of three parts (24 items), including the pain (5 items), stiffness (2 items), and joint function (17 items) [25, 26]. The higher score means the more severe symptom of osteoarthritis. Since the pain was assessed in the VAS, the modified WOMAC score in this study did not contain the pain. The modified WOMAC score was recorded during all the assessment visits (baseline and 7-, 14-, and 21-day follow-ups).

#### 2.7.3. Adverse Events

At each visit, the subjects were asked about adverse events (AEs); all AEs reports were included in the analysis. AEs mainly include allergic reactions, such as skin rashes, itching, and anaphylactoid purpura.

### 2.8. Sample Size

We estimated the sample size required for this study according to the following calculation method: *n* = 2*σ*^2^ × *f*(*α*, *β*)/(*μ*_1_ − *μ*_2_)^2^ [27, 28]. First, we evaluated that an absolute improvement of 4.7 (from *μ*_1_ to *μ*_2_) in VAS was likely the least significant clinically-relevant difference of the patients. Second, we assumed that the standard deviation of the VAS at baseline was 2.9 (*σ* = 2.9) [29]. Based on the hypothesis, we required 84 patients in two groups to have at least a 90% power (*β* = 0.1) and bilateral type I errors of 5% were excluded (*α* = 0.05). Assuming the withdrawal rate was 20%, the number of patients actually provided less than 80% power. Therefore, we recruited a total of 320 participants, 160 patients per group.

### 2.9. Statistical Analysis

Data collection and analysis were conducted according to the principles of intended treatment. Standard statistical techniques were used to characterize each group of patients. Enumeration data were compared between both groups using chi-square test. Measurement data followed normal distribution was compared with *t*-test. Measurement data followed nonnormal distribution was compared with rank sum test. Measurement data were expressed as means ± standard deviation. SAS 9.1 version was used for statistical analysis. All the statistical tests were bilateral, with a significance level of 0.05. Statistically significant differences were considered when *P* < 0.05 [30].

## 3. Results

### 3.1. General Information

**Figure 1:**
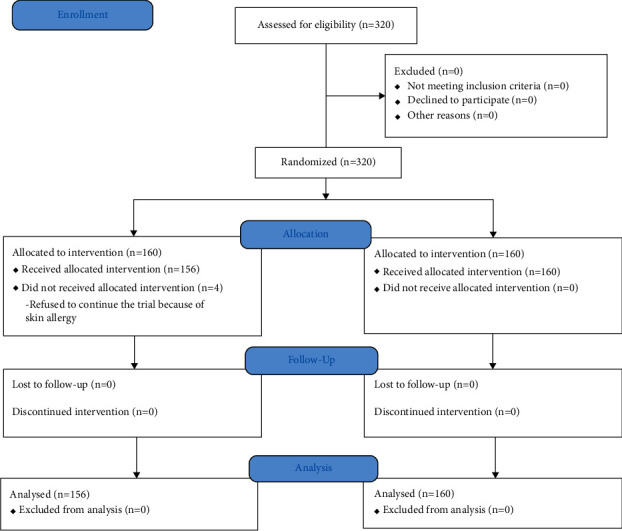
Procedure of the study.

The treatment group included 160 subjects, among which 4 withdrew from the trial. The control group also included 160 subjects, and all completed the trial. The procedure of the study is shown in Figure 1.

### 3.2. Comparison of Baseline Data

Before treatment, there was no significant difference on the distribution of gender, age, and other aspects between the two groups (*P* > 0.05). There was also no significant statistical difference in the VAS scores and WOMAC scores between two groups before treatment (*P* > 0.05).The baseline levels between both groups were coincident and comparable (Table 2).

### 3.3. Comparison at Each Time Point and Variation Trends of the VAS Scores

Normality test was first conducted in the VAS scores data of the two groups after treatment and showed that the distribution of the VAS scores data of the two groups was nonnormal at each time point (*P* < 0.05). Therefore, the data of VAS score were analyzed by rank sum test and showed that there was a significant difference (*P* < 0.05) in the VAS scores at the 7, 14, and 21 days after treatment between the two groups (Table 3).

**Figure 2:**
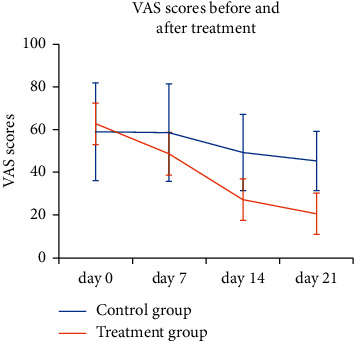
Variation trends of the VAS scores between both groups before and after the treatment.

The VAS scores at the 7, 14, and 21 days of treatment group were 22.6%, 56.5%, and 67.2% lower than pretreatment, respectively. Compared to the baseline, the VAS scores at the 7, 14, and 21 days of treatment group were significantly reduced (*P* < 0.05). The VAS scores at the 7, 14, and 21 days of control group were 0.5%, 16.4%, and 23.2% lower than pretreatment, respectively. Compared to the baseline, the VAS score of control group at the 21 days was significantly reduced (*P* < 0.05). The variation trends of the VAS scores in two groups at each time point are shown in Figure 2. There was significant difference in the variation trends of the VAS scores between both the groups (*P* < 0.05).

### 3.4. Comparison at Each Time Point and Variation Trends of the WOMAC Scores

Normality test was first conducted in the WOMAC scores data of the two groups after treatment and showed that the distribution of the WOMAC scores data of the two groups was normal at each time points (*P* < 0.05). Therefore, the data of WOMAC score at 7 and 14 days were analyzed by rank sum test and showed that there was a significant difference (*P* < 0.05) in the WOMAC scores at the 14 and 21 days after treatment between the two groups (Table 4).

**Figure 3:**
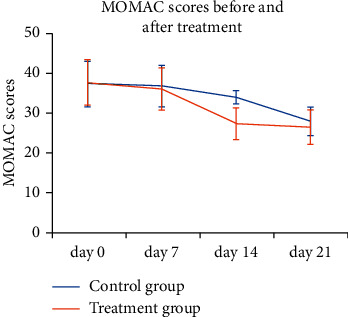
Variation trends of the WOMAC scores between both groups before and after the treatment.

The WOMAC scores at the 7, 14, and 21 days of treatment group were reduced by 4.2%, 27.2%, and 29.6%, respectively. Compared to the baseline, the WOMAC scores at the 14 and 21 days of treatment group were significantly decreased (*P* < 0.05). The WOMAC scores of control group at the 7, 14, and 21 days were reduced with 1.4%, 9%, and 25.1% changes, respectively. The variation trends of the WOMAC scores in two groups at each time point are shown in Figure 3. There was remarkable difference in the variation trends of the WOMAC scores between both the groups (*P* < 0.05).

### 3.5. Adverse Events

In the treatment group, there were 8 cases of skin allergic reaction. Among them, four cases had skin allergic reaction two weeks after using golden plaster. The symptoms were relieved after external application of Borneol cream and continued to complete the study. Two cases had skin allergic reaction one week after using golden plaster; the symptoms were relieved after external application of Borneol cream and they refused to continue to complete the study. Another 2 cases had skin allergic reaction one week after using golden plaster. Allergic symptom was relieved after treatment by a dermatologist. These 2 patients with skin allergy refused to continue to complete the study. In the control group, 2 cases had skin allergic reaction and the symptoms were relieved after external application of Borneol cream. The patients continued to complete the study. The incidence rate of AEs in the treatment group was 5%, and the control group was 1.25%. The incidence of AEs between the two groups had no statistical difference by the chi-square test (Table 5). No serious AEs were shown between both the groups.

## 4. Discussion

In ancient times, golden plaster was mainly used for traumatic wounds, such as carbuncle, boils, and sore diseases. It had shown its characteristics of easy operation and good efficacy. In recent years, golden plaster has been widely applied in the field of traumatology with good therapeutic effects after years of clinical practices. For example, the external application of golden plaster after mechanical injuries with bone fracture and soft tissues welling can inhibit aseptic inflammatory reaction, promote absorption of hematoma, improve blood circulation, and benefit the repairing of soft tissue.

Modern researches had shown that golden plaster could fast relieve swelling and pain and speed up the repair of soft tissue. There are two reasons. One is that it has the role of heat-clearing and detoxifying, which can inhibit the release and activation of peptide, histamine, and metabolite. The other is that it has the function of promoting blood circulation to remove stasis, which can effectively restrain the expansion of the capillaries and prevent the accumulation of exudate and cell infiltration resulted by the increased vascular permeability [31, 32].

At present, there were massive literatures on golden plaster, and among them, different compositions of golden plaster were used to cure different diseases [33–35], such as mammitis, orchitis, children parotitis, phlebophlogosis, herpes zoster, gouty arthritis, rheumatoid arthritis, and knee osteoarthritis. However, most of them were single-center, unblinded studies. Furthermore, randomized methods in these studies were not precise. In addition, these studies were lacking of recognized effect indicators and sufficient adverse effect reports. Therefore, a trial following the internationally recognized diagnostic criteria and outcome measurements, selecting appropriate samples and intervention time, is necessary to evaluate the treatment efficacy. In this study, here, we performed a rigorous multicenter, randomized, double-blind, placebo-controlled design. We also selected the internationally recognized outcome indicators, including pain VAS scores and WOMAC scores. For the first time, our study achieved the results of evidence-based medicine, which showed that golden plaster can effectively alleviate the pain of knee and improve the physical function in the patients with knee OA.

Golden plaster has obvious effect on alleviating pain of knee osteoarthritis, since there are the anti-inflammatory and analgesic effects of various components contained in golden plaster. Chinese rhubarb, the monarch drug in golden plaster, had been reported to have significant pharmacological effects, such as degrading inflammatory mediators in tissues and plasma, reducing tumor necrosis factor in the serum of critically ill patients, interleukin, and endotoxin levels [36, 37]. Pharmacological studies showed that, angelica root, another herb in golden plaster, had obvious antipyretic effect, analgesia, anti-inflammatory effect, and vasodilator action effect and its various effective components had the effect of relieving spasm and pain [38]. In addition, red peony root, added into golden plaster, boosts the anti-inflammatory effect, since it had the effects of anti-inflammatory, antiallergic, and scavenging active oxygen radicals [39].

Our study was still limited because the observation time was short. Since OA is a chronic degenerative disease, 3 weeks of observation is relatively short in the OA treatment scheme. Most OA clinical trials ranged from 1 month to 2 years. An ongoing trial with one year treatment plan is conducted by our team.

Overall, our study indicated that golden plaster has a significant treatment effects on the pain, stiffness, and physical function of knee OA patients in a short time. Golden plaster occasionally caused skin allergies such as rash and itching.

## Figures and Tables

**Table 1:** Prescription of golden plaster.

Herbal name	Amount (g)
Trichosanthes root	25.05
Turmeric	16.7
Angelica	16.7
Atractylodes	16.7
Licorice	16.7
Rhubarb	16.7
Phellodendron	16.7
Arisaema	16.7
Magnolia bark	16.7
Tangerine peel	16.7

**Table 2:** The baseline comparison between the control group and treatment group.

Characters	Control group	Treatment group	*P* value
Age	53.22 ± 10.85	52.69 ± 13.12	0.37
VAS scores	58.96 ± 22.78	62.79 ± 23.25	0.44
WOMAC scores	37.83 ± 5.68	38.13 ± 5.69	0.20

There was no significant difference between the control group and treatment group.

**Table 3:** The VAS scores in the two groups before and after the treatment.

Group	Case	Pretreatment	7 days after treatment	14 days after treatment	21 days after treatment
Control group	160	58.96 ± 22.78	58.69 ± 22.67	49.29 ± 17.82	45.30 ± 13.94
Treatment group	156	62.79 ± 23.25	48.61 ± 16.82^*∗*^	27.34 ± 6.83^*∗*^	20.62 ± 5.54^*∗*^

VAS = Visual Analogue Pain Scale; ^*∗*^control group VS treatment group, *P* < 0.05.

**Table 4:** The WOMAC scores in the two groups before and after the treatment.

Group	Case	Pretreatment	7 days after treatment	14 days after treatment	21 days after treatment
Control group	160	37.83 ± 5.68	37.31 ± 5.26	34.44 ± 1.66	28.33 ± 3.61
Treatment group	156	38.13 ± 5.69	36.54 ± 5.35	27.76 ± 4.01^*∗*^	26.84 ± 4.32^*∗*^

WOMAC = Western Ontario and McMaster University Osteoarthritis Index; ^*∗*^control group VS treatment group, *P* < 0.05.

**Table 5:** The incidence rate of adverse events (%).

Adverse events	Control group	Treatment group	Chi-square value	*P* value
Skin allergic reaction	1.25(2/160)	5(8/160)	3.72	0.054

There was no significant difference between the control group and treatment group.

## Data Availability

These data are owned by the Suzhou Hospital of Traditional Chinese Medicine. Access to these data will be considered by the author upon request. She can be reached at dztang702@126.com.
